# Clinical and Analytical Comparison of Monoclonal and Polyclonal Immunoassays for Fecal Pancreatic Elastase

**DOI:** 10.3390/diagnostics14111166

**Published:** 2024-05-31

**Authors:** Jasna Lenicek Krleza, Merica Aralica, Lara Milevoj Kopcinovic, Renata Zrinski Topic

**Affiliations:** 1 Department of Laboratory Diagnostics, Children’s Hospital Zagreb, 10000 Zagreb, Croatia; 2 University Department of Nursing, Catholic University of Croatia, Ilica 244, 10000 Zagreb, Croatia; 3 Department of Laboratory Medical Diagnostics, University of Applied Health Sciences Zagreb, 10000 Zagreb, Croatia; 4 Clinical Department for Laboratory Diagnostics, Clinical Hospital Centre Rijeka, 51000 Rijeka, Croatia; 5 Department of Clinical Chemistry, Sestre Milosrdnice University Hospital Center, 10000 Zagreb, Croatia; 6 School of Medicine, Catholic University of Croatia, 10000 Zagreb, Croatia

**Keywords:** exocrine pancreatic insufficiency, immunoassay, methods, pancreatic elastase

## Abstract

Background: Numerous immunoassays have been commercialized to determine pancreatic elastase (PE) in feces in screening for exocrine pancreatic insufficiency (EPI), but how the different assays compare to one another is controversial, especially in the context that all methods use the same cut-off values for interpreting the results obtained on the presence or absence of EPI or the degree of insufficiency if it is present. Our aim was to analytically verify a new method for determining PE, compare the results with a previous method, and verify the declared cut-off values for interpretation of the results. Methods: PE in the stool was assayed using a previous monoclonal enzyme-linked immunosorbent assay (“ScheBo ELISA”) and a new polyclonal particle-enhanced turbidimetric immunoassay (“Bühlmann PETIA”). The direct method comparison of two immunoassays was performed in 40 samples. Clinical comparisons were conducted against each other for the binary determination of “abnormal/normal” elastase levels and the three-way determination of “severe/moderate/no” EPI in 56 samples. The indirect comparison method used external quality assessment (EQA) data to compare the monoclonal and polyclonal immunoassays for PE, and additionally compare the monoclonal ScheBo ELISA to a monoclonal chemiluminescence immunoassay (“DiaSorin CLIA”). Results: Precision in the series and intra-laboratory precision for Bühlmann PETIA met the manufacturer’s specifications for the concentration range of limit/lower values and the range of normal values. The Bühlmann PETIA immunoassay on different analytical platforms yielded comparable results and nearly perfect agreement in the case of three-way classification (kappa = 0.89 with 95%CI from 0.79 to 1.00. ScheBo ELISA tends to generate higher values of pancreatic elastase than the Bühlmann PETIA; agreement between the methods was moderate in the case of binary classification (kappa = 0.43; 95% CI 0.25 to 0.62), and substantial in the case of three-way classification (kappa = 0.62; 95% CI 0.50 to 0.75). EQA data analysis showed a statistically significant difference between ScheBo ELISA and Bühlmann PETIA peer groups (*p* = 0.031), as well as the DiaSorin CLIA and ScheBo ELISA peer groups (*p* = 0.010). Conclusion: The ScheBo ELISA and Bühlmann PETIA do not appear to be commutable in the analytical and clinical context. Our data address a discordance between different mono- and polyclonal immunoassays for pancreatic elastase and the potential of misclassification using its universal cut-off values in screening suspected patients for exocrine pancreatic insufficiency.

## 1. Introduction

In exocrine pancreatic insufficiency, the pancreas does not produce or secrete adequate bicarbonate, enzymes such as lipase or amylase, or proteases such as elastase into the duodenum, causing maldigestion and malabsorption that can lead to weight loss, diarrhea, bloating, and steatorrhea [1]. The condition is prevalent among individuals with chronic pancreatitis, pancreatic cancer, or cystic fibrosis, and among those with a resected pancreas. The condition has also been associated with celiac disease, HIV infection, diabetes mellitus, and inflammatory bowel disease [2].

The reference method for diagnosing exocrine pancreatic insufficiency is secretin-enhanced magnetic resonance cholangiopancreatography (s-MRCP) [1]. Nevertheless, diagnosis typically involves direct and indirect pancreatic function tests (PFTs) as well as clinical examinations.

Direct PFTs require the administration of secretin or cholecystokinin to stimulate the pancreas and the measurement of enzymes in pancreatic secretions, making them invasive and impractical for patients and clinical staff [3]. “Indirect” tests do not involve stimulation of the pancreas and can be performed on blood, urine, feces, or breath. One indirect test, in which feces are analyzed for the presence of pancreatic elastase, is non-invasive and requires a small amount of stool. In contrast to a commercial test that assays chymotrypsin in stool, assays of pancreatic elastase are unaffected by pancreatic enzyme replacement therapy, a common treatment in exocrine pancreatic insufficiency. Other indirect PFTs have the disadvantages of lacking standardization or showing poor sensitivity for pancreatic exocrine dysfunction [3].

Pancreatic elastase is recommended as part of the diagnostic work-up in individuals suspected of having exocrine pancreatic insufficiency because it correlates well with s-MRCP, although its sensitivity is modest [4]. In stool, pancreatic elastase complexes with sterols, protecting it from degradation during sampling and transport to the laboratory [5,6].

Various types of immunoassays have been commercialized to detect pancreatic elastase in feces. The immunoassays differ in how the enzyme is extracted from stool, whether mono- or polyclonal antibodies are involved, and whether the analytical method relies on enzyme-linked immunosorbence, particle-enhanced turbidity, or chemiluminescence [7,8]. How these various assays compare to one another is incompletely understood, perhaps because some of them have only recently entered the market. Such comparisons are sorely needed given that the few studies available suggest potentially important analytical and clinical discordance, yet clinical guidelines continue to propose universal cut-off values independent of the type of immunoassay [4,8,9].

New methods require a verification procedure before being introduced into routine laboratory work. In our study, after the successful analytical verification procedure of the new immunoturbidimetric method (hereafter the “Bühlmann PETIA”) in the Children’s Hospital Zagreb laboratory, a comparison was performed with the previous method (hereafter the “ScheBo ELISA”).

The initial results of the comparability of these two methods (ScheBo ELISA vs. Bühlmann PETIA) did not provide satisfactory results, and the comparison of the methods was extended considerably. The significance of the comparability or non-comparability of the methods refers to the cut-off values, which are identical for both methods, according to the declaration of the reagent manufacturer [10,11]. For this reason, this study aimed to investigate the comparability of the different methods and the validity of using the same cut-off values, including the results of our extended research.

Here, we compared two immunoassays for fecal pancreatic elastase that differed in stool extraction protocol, antibody type, and assay format: one was a recently launched semi-automated particle-enhanced turbidimetric immunoassay involving a polyclonal antibody (“Bühlmann PETIA”); the other is a well-established enzyme-linked immunosorbent assay involving a monoclonal assay (ScheBo ELISA) [10,11]. We are unaware of analytical or clinical comparisons between the ScheBo ELISA and Bühlmann PETIA. Here, we performed a direct analytical comparison of the two methods against each other using our own data, and we drew on external quality assessment (EQA) data for indirect, independent verification of our findings. Furthermore, we conducted a clinical comparison of ScheBo ELISA and Bühlmann PETIA. Finally, using the EQA data, we compared the ScheBo ELISA against the novel semi-automated monoclonal chemiluminescence immunoassay (hereafter the “DiaSorin CLIA”). The goal of this study was to provide insights into potential disagreement between the two monoclonal immunoassays for pancreatic elastase. We focused on the Bühlmann PETIA and DiaSorin CLIA as two novel immunoassays because they are suitable for automation on an analyzer, which may lead to faster results than the time-consuming ScheBo ELISA.

## 2. Materials and Methods

This cross-sectional study involved three tertiary healthcare centers: the Children’s Hospital Zagreb (CHZ), Sestre milosrdnice University Hospital Center (SM UHC) and the Clinical Hospital Center Rijeka (CHCR), all located in Croatia.

### 2.1. Study Subject and Samples

Stools for this prospective observational study were obtained between 1 March 2021 and 30 November 2023 from a convenience sample of patients at Children’s Hospital Zagreb (Zagreb, Croatia). At the reception, the only inclusion criterion for samples was that they be formed feces of patients referred for routine analysis of pancreatic elastase. The study design was approved by the Ethics Committees of Children’s Hospital Zagreb, and the study was performed in accordance with the Declaration of Helsinki.

Only 56 of 4926 stool samples (1.1%) at the Children’s Hospital Zagreb were analyzed because, in accordance with CLSI standards, we limited our analysis to samples in which the values of fecal pancreatic elastase were within the determination range of the analytical methods (ELISA: 15 to 500 µg/g and PETIA: 10 to 500 µg/g). Although the measuring ranges of both methods are almost identical, due to the difference in the measured concentrations with both methods, a small number of samples met the specified CLSI criteria. For example, only the concentrations of FE > 10 µg/g and <300 µg/g determined with the PETIA method fulfilled the CLSI criterion of the measuring range for both methods.

The subset of stools was stored at 2–8 °C for up to one day during transport from Children’s Hospital Zagreb to the Clinical Hospital Center Rijeka. Samples were frozen at −20 °C upon arrival in the Clinical Hospital Center Rijeka. Sample storage and transport conditions were considered sufficient to maintain the stability of pancreatic elastase based on manufacturer-reported data [10,11].

Additional analysis was performed in the extracts of pancreatic elastase by the CALEX Cap devices, from the CHZ. In total, 40 liquid extracts of patient stool samples were sent in the CALEX Cap devices from the CHZ to the SM UHC for the analysis. After reception, all extracts were stored at −20 °C for up to 5 days. According to the manufacturer, the extracts are stable at −20 °C for up to 3 months when stored within the devices (B-CALEX). In the SM UHC, the analysis was performed using the Buhlmann fPELA turbo kit at the Architect c4000, (Abbott, IL, USA) instrument.

One rationale for extra analysis reflects the need for clinical laboratory staff to provide evidence of potential data bias if the PE extracts by CALEX Cap devices are processed by the same immunoassay (Bühlmann PETIA) on two different platforms (AU 860 and Architect c4000).

### 2.2. Analytical Verification of the Bühlmann PETIA Method

From March to August 2021, analytical verification of the new Bühlmann PETIA method for the determination of pancreatic elastase in stool was carried out in the Medical and Biochemical Laboratory of the Children’s Hospital Zagreb. The verification procedure was planned in accordance with the recommendations of the Working Group on Measurement Uncertainty of the Croatian Society of Medical Biochemistry and Laboratory Medicine and the Croatian Chamber of Medical Biochemists [12] and included the determination of precision in series and intra-laboratory precision.

In the verification process, commercial controls of the manufacturer were used in two levels (Bühlmann: B-KPELA-CONSET, Lot: 3307, Level 1: average: 150 μg/g with range: 120 to 180 μg/g and Level 2: average: 400 μg/g with range: 320 to 480 μg/g), in a short period (five days in triplicate, same lot of reagents and calibrators, and the same operator under the same conditions) and the results were compared with the defined criteria of and acceptable range of variation by the manufacturer (for precision in series, coefficient of variation, CV, is <15% and for intra-laboratory precision, CV is <10%).

### 2.3. Direct Comparison of ScheBo ELISA and Bühlmann PETIA

At Children’s Hospital Zagreb, pancreatic elastase was extracted from patient stool samples using a CALEX^®^ Cap device (Bühlmann Laboratories, Schönenbuch, Switzerland) and analyzed using the Bühlmann fPELA^®^ Turbo system (Bühlmann Laboratories) on an AU 680 analyzer (Beckman Coulter Diagnostics, Brea, CA, USA) on the same day as when samples arrived at the clinical laboratory [10,13].

At the Clinical Hospital Center Rijeka, pancreatic elastase was extracted from stool samples using the ScheBo Master Quick-Prep^®^ device (ScheBo Biotech AG, Giessen, Germany) and analyzed using the ScheBo Pancreatic Elastase 1 Stool Test (catalog no. 07, ScheBo Biotech AG), as described [11,14]. In these cases, pancreatic elastase was extracted from stool samples that had been stored up to 60 days at −20 °C. Analysis of batch samples was performed on the ThunderBolt ELISA analyzer (Gold Standard Diagnostics, Davis, CA, USA).

Levels of pancreatic elastase below the low level of linearity, corresponding to levels < 10 μg/g in the Bühlmann PETIA or <15 μg/g in the ScheBo ELISA, were recorded as 10 or 15 μg/g in the final data analysis.

The two methods were compared in terms of analytical performance, as described in document EP15-A3 of the Clinical and Laboratory Standards Institute [15]. The two methods were also compared in terms of clinical performance by translating measured levels of pancreatic elastase into a binary classification of “abnormal pancreatic elastase level” (<200 µg/g) or “normal pancreatic elastase level” (>200 µg/g), or into a three-way classification of “severe exocrine pancreatic insufficiency” (<100 μg/g), “moderate exocrine pancreatic insufficiency” (100 to 200 μg/g), or “no exocrine pancreatic insufficiency” (>200 μg/g). The binary cut-off reflects the threshold frequently used in the clinic, while the three-way cut-offs are recommended by the studied manufacturers [4].

### 2.4. Indirect Comparisons of Immunoassays Using External Quality Assessment (EQA) Data

For indirect comparison of immunoassays, we used data for fecal pancreatic elastase from the feces diagnostics (FD) scheme of the Reference Institute for Bioanalytics (RfB; Bonn, Germany), in which the Children’s Hospital Zagreb participated [16]. In the FD scheme from 2020 to 2023, there were eight rounds with two lyophilized human stool samples per round (samples A and B), corresponding to 16 samples in total. The participants’ results in both samples per round were reported as medians and ranges in the corresponding method-related (“peer”) groups. The Children’s Hospital Zagreb reported its results in the Bühlmann PETIA peer group.

Medians from the ScheBo ELISA, Bühlmann PETIA, and DiaSorin CLIA (DiaSorin S.p.A, Saluggia, Italy) peer groups were selected for the statistical analysis only if all medians from samples A or B per round from these three peer groups exceeded the lower level of linearity of the single immunoassays. This was the case in 13 out of 16 samples.

Finally, we compared the ScheBo ELISA peer group pairwise with the Bühlmann PETIA and DiaSorin CLIA peer groups.

### 2.5. Clinical Comparison

The clinical comparison of different diagnostic tests for the determination of PE in stool is based on the correct categorization of the results obtained, classified into three classes with a description of pancreatic function: (1) severe insufficiency (PE < 100 μg/g), (2) moderate insufficiency (PE = 100 to 200 μg/g), and (3) no insufficiency (PE > 200 μg/g). It is important to emphasize that the cut-off value (200 μg/g) and the range of PE for the PI categories are the same for all methods (declared by manufacturer).

Considering that pancreatic elastase is a screening test for PI, coming before other diagnostic tests (mainly invasive), its results may lead to further investigation or to its cancelation. In other words, if one assay overestimates the PE value, its normal results (>200 μg/g ug/g) may stop any further testing and lead to incorrect conclusions about the presence of disease.

In this context, it is important to determine the agreement of the results by different methods according to the declared values for the interpretation of the results, i.e., to perform a clinical comparison.

### 2.6. Statistical Analysis

Data were analyzed statistically using MedCalc 20.008 (MedCalc Software, Ostend, Belgium), and differences associated with *p* < 0.05 were considered significant.

The Kolmogorov–Smirnov test was used for assessment of the normality of data distribution. Absolute and relative mean differences between ScheBo ELISA and Bühlmann PETIA were compared using Bland–Altman analysis and Passing–Bablok regression.

The ScheBo ELISA and Bühlmann PETIA were compared clinically by calculating the kappa coefficient of inter-rater agreement according to the binary and three-way classifications in Section 2.1. The level of agreement was classified as follows: kappa < 0.0, poor; kappa between 0.0 and 0.20, slight; kappa between 0.21 and 0.40, fair; kappa between 0.41 and 0.60, moderate; kappa between 0.61 and 0.80, substantial; and kappa > 0.80, nearly perfect. Kappa coefficients were reported together with their 95% confidence interval (95%CI).

Differences between two peer groups were assessed for significance using the Mann–Whitney test (U test) if at least one of the groups in the comparison showed a skewed distribution.

## 3. Results

### 3.1. Analytical Verification of the Bühlmann PETIA method

The results of the analytical verification, which include the determination of precision in series and intra-laboratory precision, are presented in Table 1.

The obtained results of the series and intra-laboratory precision met the manufacturer’s specifications for the concentration range of limit/lower values and the range of normal values.

### 3.2. Data Agreement at Two Different Platforms (Pancreatic Elastase in Shared Extracts)

**Figure 1:**
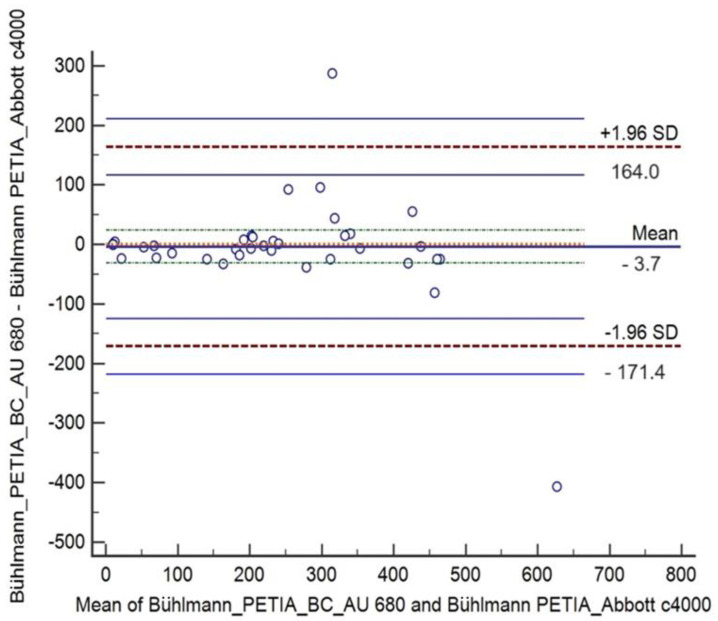
Bland–Altman analysis of Bühlmann PETIA for determining pancreatic elastase in stool samples at AU 860 and Architect c4000: absolute mean difference (−3.7 μg/g) with 95%CI (green dot line above and below absolute mean) and limits of agreement (+/−1.96 SD, red dot line) with 95%CI (blue line above and below of limits of agreement).

**Figure 2:**
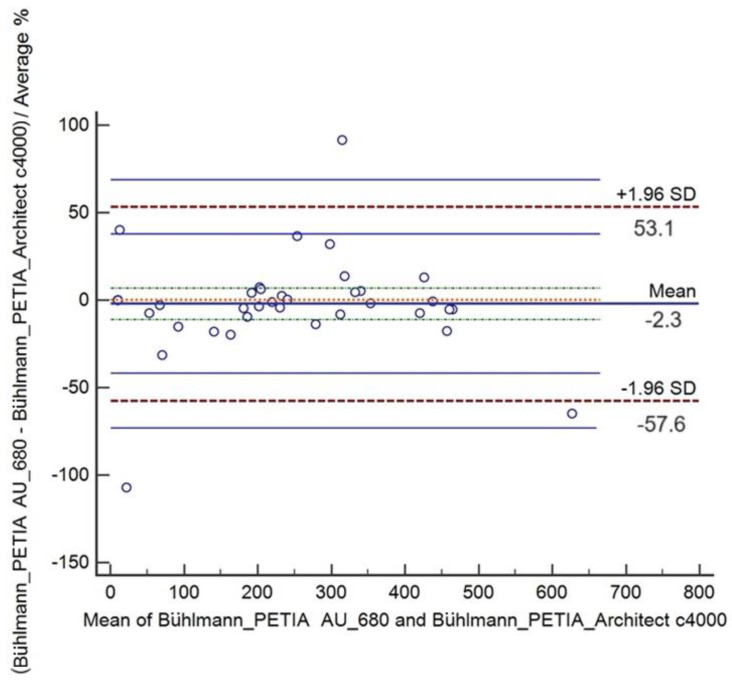
Bland–Altman analysis of Bühlmann PETIA for determining pancreatic elastase in stool samples at AU 860 and Architect c4000: relative mean difference (−2.3%) with 95%CI (green dot line above and below relative mean) and limits of agreement (+/−1.96 SD, red dot line) with 95%CI (blue line above and below of limits of agreement).

The absolute Bland and Altman plot showed the mean differences between PE extract results (N = 40) by the Bühlmann PETIA at AU 860 and Architect c4000 that was negative −3.7 μg/g with a 95% CI of −31.06 to 23.66 and agreement ranging from −171.4 μg/g to 164.0 μg/g (Figure 1). In line with the absolute differences, the relative Bland and Altman plot showed a negative mean of −2.3% μg/g with a 95%CI of −11.30 to 6.76 and agreement ranging from −57.6% to 53.0% (Figure 2).

Graphical and numerical data from both analyses showed a lack of bias between the two analytical platforms in the performance of Bühlmann PETIA as the line of equality is within 95% CI of the mean difference. However, a few points were out of the intervals but still in the agreement limits unlike the two points (differences) that were outliers.

**Figure 3:**
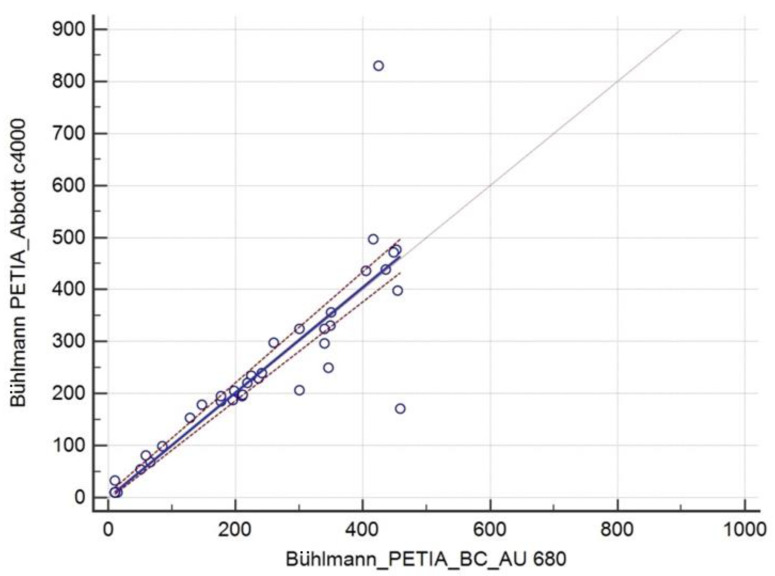
Passing–Bablok regression analysis of the pancreatic elastase from shared extracts assessed by Bühlmann PETIA at AU 860 and Architect c4000. The solid line corresponds to the regression analysis equation; dashed lines correspond to the 95%CI of the intercept and slope.

Passing–Bablok analysis revealed regression equation y = −0.10 + 1.00x with a 95%CI for the intercept of −3.70 to 8.80 and a 95%CI for the slope of 0.95 to 1.06. The Cusum test for linearity was *p* = 0.17 (Figure 3).

Inter-rater agreement analysis of Bühlmann PETIA data from AU 860 and Architect c4000 (N = 40; cut-off < 200 μg/g (category 1, abnormal) and cut-off > 200 μg/g (category 2, normal) showed a weighted kappa coefficient of 0.80 with a 95%CI from 0.62 to 0.99.

Inter-rater agreement analysis of Bühlmann PETIA data from AU 860 and Architect c4000 (N = 40; cut-off <100 μg/g (category 1), cut-off 100 to 200 μg/g (category 2), and cut-off > 200 μg/g (category 3) revealed a nearly perfect weighted kappa coefficient of 0.89, with a 95%CI from 0.79 to 1.00 (Table 2).

### 3.3. Direct Comparison of ScheBo ELISA and Bühlmann PETIA

**Figure 4:**
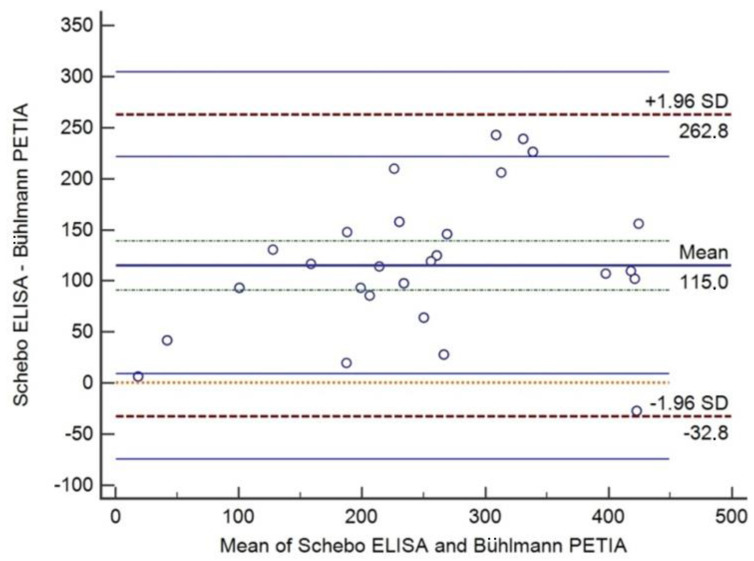
Bland–Altman analysis between the ScheBo ELISA and Bühlmann PETIA for determining pancreatic elastase in stool samples: absolute mean difference (115.0 μg/g) with the 95%CI (green dot line above and below absolute mean) and limits of agreement (+/−1.96 SD, red dot line) with 95%CI (blue line above and below of limits of agreement).

**Figure 5:**
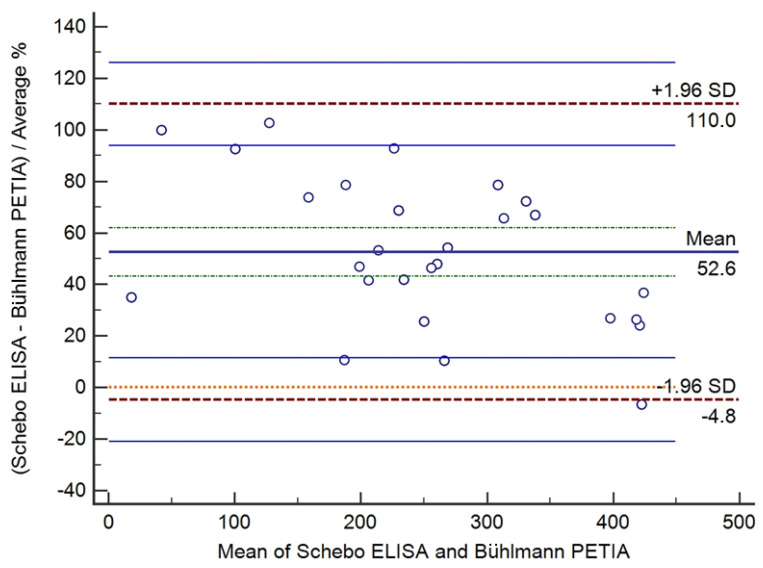
Bland–Altman analysis between the ScheBo ELISA and Bühlmann PETIA for determining pancreatic elastase in stool samples: relative mean difference (−52.6%) with the 95%CI (green dot line above and below relative mean) and limits of agreement (+/−1.96 SD, red dot line) with the 95%CI (blue line above and below of limits of agreement).

Based on Bland–Altman analysis of the pancreatic elastase determinations of 40 samples, the absolute mean difference between the ScheBo ELISA and Bühlmann PETIA was +115.0 μg/g (95% CI 90.9 μg/g to 139.1 μg/g) and the range of agreement was −32.8 μg/g to 262.8 μg/g (Figure 4). The relative mean difference was 52.6% (95% CI 43.2% to 62.0%) and the range of agreement was −4.8% to 110.0% (Figure 5).

As shown in both figures, mean differences and corresponding 95%CIs lay above the line of equality, indicating positive trends of ScheBo ELISA measurements of pancreatic elastase in respect to Bühlmann PETIA as well as significant bias between the results of the two methods. In other words, the ScheBo ELISA tended to significantly overestimate pancreatic elastase with respect to the Bühlmann PETIA.

**Figure 6:**
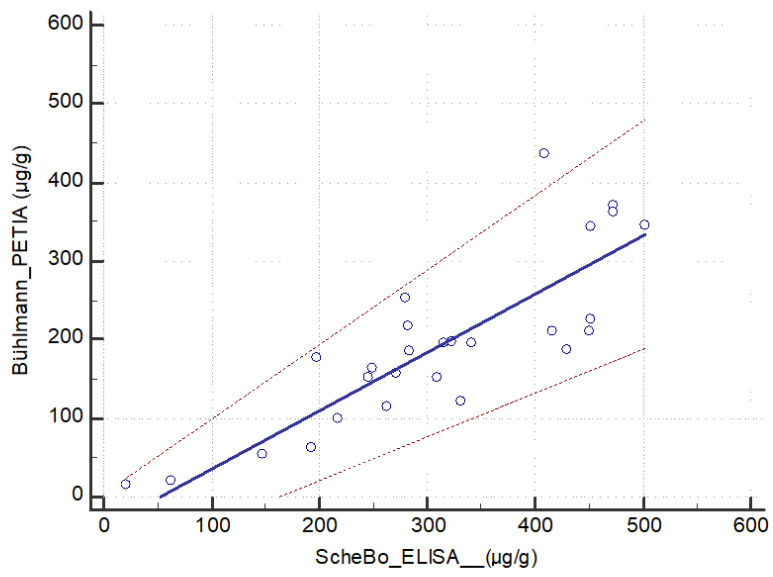
Passing–Bablok regression to compare the ScheBo ELISA and Bühlmann PETIA for determining fecal pancreatic elastase. The solid line corresponds to the line of best fit, while the dashed lines indicate the 95% confidence intervals of the slope and intercept.

Passing–Bablok regression of the determinations for the two immunoassays yielded the line of best fit equation of y = −40.8 + 0.8x, where the 95%CI for the intercept was −92.6 to 3.9 and the 95% CI for the slope was 0.56 to 0.94, with no significant deviation from linearity (*p* = 0.30; Figure 6). These results support the idea that the ScheBo ELISA tends to generate higher values of pancreatic elastase than the Bühlmann PETIA.

Next, we clinically compared the two immunoassays by assessing their agreement in classifying categories of “abnormal/normal” levels of pancreatic elastase or “no/moderate/severe” exocrine pancreatic insufficiency. Agreement between the methods was moderate in the case of binary classification, with a kappa of 0.43 (95%CI 0.25 to 0.62; Table 3); and substantial in the case of three-way classification, with a kappa of 0.62 (95%CI 0.50 to 0.75; Table 4).

### 3.4. Indirect Comparisons of Immunoassays Using EQA Data

**Figure 7:**
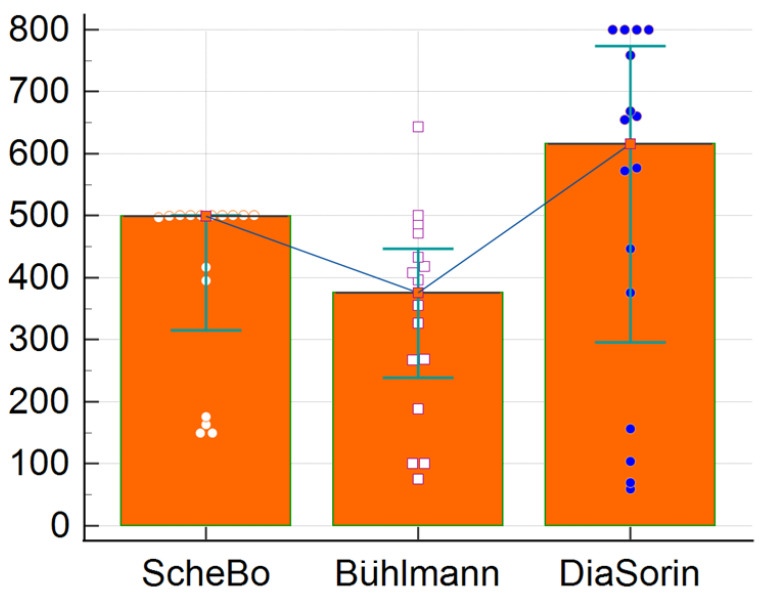
Comparison of the ScheBo ELISA (white circles), Bühlmann PETIA (white squares), and DiaSorin CLIA (blue cicles) peer groups in external quality assessment data in feces diagnostics for pancreatic elastase. Multivariable graph with peer group medians (orange squers) in all rounds from 2020 to 2023 and for both sample types (Sample A and Sample B), with error bars (95%CI of median) (green bars) and trend lines (the blue line connecting the peer group medians).

Median determinations of pancreatic elastase in selected ScheBo ELISA, Bühlmann PETIA, and DiaSorin CLIA peer groups in both samples of the “Feces Diagnostics” EQA scheme run by the Reference Institute for Bioanalytics showed that the median in the ScheBo ELISA peer group was significantly higher than the median in the Bühlmann PETIA peer group (*p* = 0.014). Additionally, the median DiaSorin CLIA peer group was significantly higher than the ScheBo ELISA peer group (*p* = 0.003; Table 5 and Figure 7).

## 4. Discussion

After the successful analytical verification and comparison of pancreatic elastase results of patients determined by the same immunoassay (Bühlmann PETIA) at two different platforms (AU 860 and Architect c4000), the focus of this study was a comparison of two immunoassays for fecal pancreatic elastase that differed in stool extraction protocol, antibody type, and assay format, but with the same cut-off values: one was a recently launched semi-automated particle-enhanced turbidimetric immunoassay involving a polyclonal antibody (“Bühlmann PETIA”); the other was a well-established enzyme-linked immunosorbent assay involving a monoclonal assay (ScheBo ELISA). In addition, comparison with the monoclonal immunoassay (DiaSorin CLIA) was performed using an indirect method (using EQA data).

Our study provides analytical and clinical comparisons of the well-established monoclonal ScheBo ELISA and a novel polyclonal Bühlmann PETIA, two immunoassays accepted for routine clinical analysis. Our findings from the Bland–Altman analysis suggest that the ScheBo ELISA tends to overestimate the enzyme fecal concentration relative to the Bühlmann PETIA. In fact, the absolute mean difference between ScheBo ELISA and Bühlmann PETIA was 115.0 μg/g, and the range of agreement had an upper limit of 262.8 μg/g, entering the range of normal values. Our findings from the Passing–Bablok regression support the idea that the ScheBo ELISA tends to generate higher values of pancreatic elastase than the Bühlmann PETIA. These results, considering the widespread use of 200 μg/g for the classification of individuals with normal or abnormal levels of pancreatic elastase during screening for exocrine pancreatic insufficiency, suggest that the two immunoassays are not commutable in an analytical context.

While several studies have suggested that immunoassays for pancreatic elastase differ in performance depending on whether they involve mono- or polyclonal antibodies, other work has suggested no significant difference [8,9,17]. The extensive comparison of five immunoassays based on EQA data showed a lack of agreement between mono- or polyclonal immunoassays for pancreatic elastase at levels < 200 μg/g (considered suggestive of exocrine pancreatic insufficiency) and for levels > 200 μg/g (outside clinical interest) [18]. We also detected discordance in the EQA data not only between mono- and polyclonal immunoassays (ScheBo ELISA vs. Bühlmann PETIA), but also between two monoclonal immunoassays (ScheBo ELISA vs. DiaSorin CLIA). This indirect discordance between Bühlmann PETIA vs. ScheBo ELISA supports our findings from their direct comparison, making the correct clinical conclusions difficult without consideration of the patient’s clinical manifestation at the sampling time.

Indeed, in the context of clinical agreement between ScheBo ELISA and Bühlmann PETIA, we found only moderate to substantial agreement for classifying individuals as showing “abnormal” or “normal” levels of pancreatic elastase, or as having “no”, “moderate”, or “severe” exocrine pancreatic insufficiency. Up to one-third of individuals may be misclassified by the ScheBo ELISA because of its overestimation of pancreatic elastase. This may lead to their exclusion from screening, with potentially unfavorable outcomes such as delaying proper diagnosis and treatment. Considering the published literature on clinical agreement between immunoassays for pancreatic elastase, there is an inconsistency in statistical analysis, limiting comprehensive understanding of the issue.

The accurate detection of pancreatic elastase in stool can strongly depend on how it is extracted from stool, which is generally performed using commercial devices. However, the extraction of watery samples may cause dilution effects, resulting in falsely positive PE, regardless of the type of extraction. For solving these issues, the lyophilization of watery stool has been suggested [19]. In our study, two types of extraction devices were used for formed stools, each related to a corresponding manufacturer. We did not compare the two extraction devices to each other because that lay outside the scope of our study. However, it cannot be neglected that different extraction devices and associated protocols, even in formed stools, may carry a risk of bias.

Another potential source of discordance between studied immunoassays may be the type and origin of associated calibrators. In our study, both immunoassays used calibrators based on recombinant human pancreatic elastase, but only the Bühlmann stated that the calibrators are standardized against an internal reference material. The lack of reference standards for pancreatic elastase results in the use of manufacturer’s specific reference material and prevents harmonization between immunoassays.

Furthermore, the ELISA method, if performed manually and in uncontrolled conditions, has many factors that can affect the results (for example, changes in temperature, humidity, precise time, preparation, and the storage of solutions) compared to automated methods (CLIA and PETIA). In our study, ELISA was performed using ThunderBolt (Gold Standard Diagnostics, Davis, CA, USA), an ELISA processor, making all contributing factors highly controlled.

The major limitation of our study is related to the modest number of included stools; the selection of 40 samples is in accordance with a requirement in the CLSI 15-A3 document for user verification and estimations of bias [15]. In a developing process of this study, we included DiaSorin CLIA data from an EQA FD scheme, instead of directly testing the collected stools using that immunoassay. Indirect method comparison may be a shortcoming, as could the modest number of included rounds from the EQA. Currently, it seems that two or three rounds per year are commonly provided in the European EQA FD schemes [16,18]. However, the EQA approach enables insights in PE ranges of distributed samples, showing predominance those with no clinical value. Furthermore, from EQA data over a course of time, a decrease in the total number of participants in peer groups is obvious with time-consuming immunoassays. For that reason, our results should be verified with larger numbers of samples and through direct comparison with the DiaSorin CLIA method, instead of the indirect approach that we used here. Our analysis of EQA data highlights the need for more rounds with pancreatic elastase levels in areas of clinical relevance. Our study justifies more extensive direct and indirect comparisons among commercial immunoassays for pancreatic elastase. As EQA databases grow, it may become easier to detect potential discordance between assays through indirect comparisons, which can be verified through direct comparisons.

Based on our results, we can conclude that the new, commercial polyclonal immunoassay test (Bühlmann PETIA) shows excellent analytical characteristics, as well as the comparability of the results obtained by this method on different analytical platforms (Beckman Coulter AU 680 vs. Abbott Architect c4000). However, the comparability of the results of this method with other types of monoclonal immunoassays (ScheBo ELISA, DiaSorin CLIA) did not show satisfactory results, especially in the context that all methods use the same cut-off values for interpreting the results obtained on the presence or absence of pancreatic insufficiency, and the degree of insufficiency if it is present. Using the indirect method, the incomparability of the results was observed not only between mono- and polyclonal immunoassays, but also between different monoclonal immunoassays. Based on these results, we can conclude that it is necessary to consider this discrepancy when interpreting the results and the risk due to the inappropriate application of universal cut-off values when screening for exocrine pancreatic insufficiency.

## Figures and Tables

**Table 1:** Our analytical verification results, including the determination of series and intra-laboratory precision, and compared to the manufacturer’s results and criteria.

Precision in Series					
B-KPELA-CONSET (Bühlmann) Lot: 3307 Level 1 (average: 150 μg/g; range: 120–180 μg/g)	1st day	2nd day	3rd day	4th day	5th day
Measurement 1	153	159	156	148	154
Measurement 2	159	145	153	152	155
Measurement 3	151	152	156	140	153
Arithmetic mean	154	152	155	147	154
Standard deviation for each series	4.2	7.0	1.7	6.1	1.0
Pooled standard deviation for 5 days			4.0		
CV% Our result According to the manufacturer			2.6 1.9 to 6.2 (<15 *)		
**Validation of intermediate precision in repeated measurements**					
Average of the 5-day measurements			152		
Validation of intermediate precision in repeated measurements			2.9		
CV% Our result According to the manufacturer			1.09 /		
**Intra-laboratory precision**					
Intra-laboratory standard deviation					
CV% Our result According to the manufacturer			3.3 0.9 to 2.2 (<10 *)		
**Precision in series**					
B-KPELA-CONSET (Bühlmann) Lot: 3307 Level 2 (Average: 400 μg/g; range: 320–480 μg/g)	1st day	2nd day	3rd day	4th day	5th day
Measurement 1	412	407	406	393	383
Measurement 2	399	408	389	404	398
Measurement 3	402	400	400	402	398
Arithmetic mean	404	405	398	400	393
Standard deviation for each series	7	4	9	6	9
Pooled standard deviation for 5 days			7		
CV % Our result According to the manufacturer			1.75 1.9 to 6.2 (<15 *)		
**Validation of intermediate precision in repeated measurements**					
Average of the 5-day measurements			400		
Validation intermediate precision in repeated measurements			4		
CV% Our result According to the manufacturer			1.0 /		
**Intra-laboratory precision**					
Intra-laboratory standard deviation			2.4		
CV% Our result According to the manufacturer			0.6 0.9 to 2.2 (<10 *)		

Legend: (*)—manufacturer criterion of acceptable range of variation; CV—coefficient of variation.

**Table 2:** Inter-rater agreement analysis of Bühlmann PETIA data from AU 860 and Architect c4000.

Bühlmann PETIA from Architect c4000	Bühlmann PETIA from AU 860				Agreement between the Methods: Three-Way Classification
	Severe Insufficiency	Moderate Insufficiency	No Insufficiency	Total	
Severe insufficiency	12	0	0	12	Nearly perfect: kappa coefficients: 0.89 (95%CI 0.79 to 1.00)
Moderate insufficiency	0	5	3	8	
No insufficiency	0	1	19	20	
Total	12	6	22	40	

Legend: Severe insufficiency—pancreatic elastase < 100 μg/g; moderate insufficiency—pancreatic elastase between 100 and 200 μg/g; no insufficiency—pancreatic elastase > 200 μg/g; all values are numerical (*n*).

**Table 3:** Agreement in binary clinical classification between the ScheBo ELISA and Bühlmann PETIA.

Bühlmann PETIA	ScheBo ELISA			Agreement between the Methods: Binary Classification
	Abnormal	Normal	Total	
Abnormal	24	17	41	Moderate kappa coefficients 0.43 (95% CI 0.25 to 0.62)
Normal	0	15	15	
Total	24	32	56	

Legend: Abnormal—pancreatic elastase < 200 μg/g; normal—pancreatic elastase > 200 μg/g; all values are numerical (*n*).

**Table 4:** Agreement of three-way clinical classification between the ScheBo ELISA and Bühlmann PETIA.

Bühlmann PETIA	ScheBo ELISA				Agreement between the Methods: Three-Way Classification
	Severe Insufficiency	Moderate Insufficiency	No Insufficiency	Total	
Severe insufficiency	19	3	0	22	Substantial kappa coefficients: 0.62 (95% CI 0.50 to 0.75)
Moderate insufficiency	0	1	18	19	
No insufficiency	0	0	15	15	
Total	19	4	33	56	

Legend: Severe insufficiency—pancreatic elastase < 100 μg/g; moderate insufficiency—pancreatic elastase between 100 and 200 μg/g; no insufficiency—pancreatic elastase > 200 μg/g; all values are numerical (*n*).

**Table 5:** Comparison of the ScheBo ELISA, Bühlmann PETIA, and DiaSorin CLIA peer groups in the external quality assessment data.

Testing Round (Year) 2020–2023	Median Pancreatic Elastase (μg/g)					
	Sample A			Sample B		
	Peer Groups					
	ScheBo (ELISA)	Buhlmann (PETIA)	DiaSorin (CLIA)	ScheBo (ELISA)	Buhlmann (PETIA)	DiaSorin (CLIA)
FD1/20	395	268	376	15.0	10,0	10.4
FD2/20	17.6	10.0	7.0	500	643	800
FD1/21	499	397	668	500	433	800
FD2/21	417	267	447	15.0	7.5	5,9
FD1/22	500	487	800	500	418	577
FD2/22	500	408	572	497	327	655
FD1/23	16.3	18.9	15.6	500	472	759
FD2/23	500	500	800	500	355	661

Legend: FD—feces diagnostics.

## Data Availability

Original data can be obtained from Jasna Lenicek Krleza upon request.
